# Detection of Mutations Affecting Heterogeneously Expressed Phenotypes by Colony Immunoblot and Dedicated Semi-Automated Image Analysis Pipeline

**DOI:** 10.3389/fmicb.2017.02044

**Published:** 2017-10-20

**Authors:** Erik Bakkeren, Tamas Dolowschiak, Médéric R. J. Diard

**Affiliations:** ^1^Department of Biology, Institute of Microbiology, ETH Zürich, Zürich, Switzerland; ^2^Institute of Experimental Immunology, University of Zürich, Zürich, Switzerland

**Keywords:** evolution, virulence, code: ImageJmacro, *Salmonella Typhimurium*, bimodality, clonal diversity, colony immunoblot, phenotypic heterogeneity

## Abstract

To understand how bacteria evolve and adapt to their environment, it can be relevant to monitor phenotypic changes that occur in a population. Single cell level analyses and sorting of mutant cells according to a particular phenotypic readout can constitute efficient strategies. However, when the phenotype of interest is expressed heterogeneously in ancestral isogenic populations of cells, single cell level sorting approaches are not optimal. Phenotypic heterogeneity can for instance make no-expression mutant cells indistinguishable from a subpopulation of wild-type cells transiently not expressing the phenotype. The analysis of clonal populations (e.g., isolated colonies), in which the average phenotype is measured, can circumvent this issue. Indeed, no-expression mutants form negative populations while wild-type clones form populations in which average expression of the phenotype yields a positive signal. We present here an optimized colony immunoblot protocol and a semi-automated image analysis pipeline (ImageJ macro) allowing for rapid detection of clones harboring mutations that affect the heterogeneous (i.e., bimodal) expression of the Type Three Secretion System-1 (TTSS-1) in *Salmonella enterica* serovar Typhimurium. We show that this protocol can efficiently differentiate clones expressing TTSS-1 at various levels in mixed populations. We were able to detect the emergence of *hilC* mutants in which the proportion of cells expressing TTSS-1 was reduced compared to the ancestor. We could also follow changes in the frequency of different mutants during long-term infections. This demonstrates that our protocol constitutes a tractable approach to assess semi-quantitatively the evolutionary dynamics of heterogeneous phenotypes, such as the expression of virulence genes, in bacterial populations.

## Introduction

Investigating microorganisms’ evolution in action is instrumental to grasp the fundamental mechanisms and dynamics of adaptation (Lenski, 2017). From a practical point of view, e.g., in pathogenic microorganisms, rapid within-host microevolution can dramatically affect virulence, evasion of host immune defenses, increased competitiveness against species occupying the same niche, and resistance to antibiotics or predator grazing (Bliven and Maurelli, 2016; Didelot et al., 2016; Diard and Hardt, 2017). A better understanding of the evolutionary dynamics of pathogens should inspire innovative strategies to manage virulence, and prevent or treat diseases.

Whole-genome deep-sequencing can detect variants in evolving populations of microorganisms (Mcelroy et al., 2014; Didelot et al., 2016). It may, however, prove difficult to link genetic variations to phenotypic changes and to comprehend the mechanism underlying their relative effect on the fitness of organisms living in dynamic and complex environments, such as pathogenic bacteria in their hosts. Moreover, errors can occur at many sample processing steps, which can lead to false-positive signals (Mcelroy et al., 2014). If tools nevertheless exist limiting this issue, whole-genome deep-sequencing remains a time-consuming process, requiring advanced bioinformatics pipelines that are not always readily available (Kojima et al., 2016).

**FIGURE 1:**
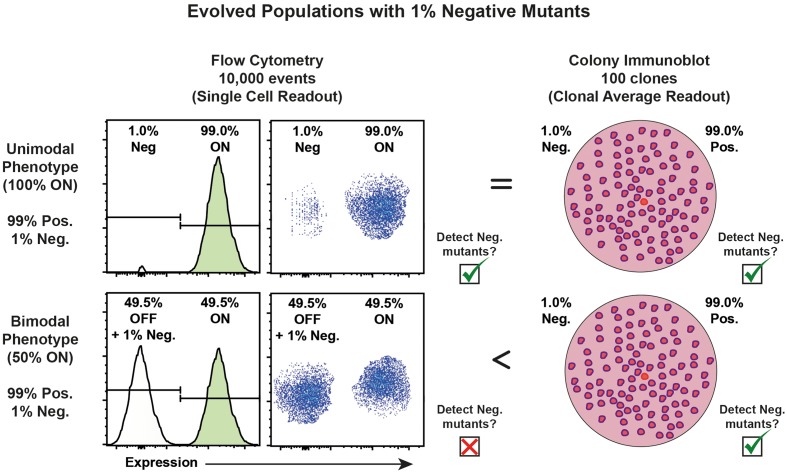
Single cell level vs. colony level phenotypic detection of mutants. For a narrow distribution of the wild-type expression of a phenotype, single cell level analysis (e.g., by flow cytometry) and colony level screening could both detect no-expression mutants. However, if only 100 clones can be screened on one membrane, mutants present at lower frequency than about 1% are difficult to detect by immunoblot. Thousands of cells can, however, be screened by flow cytometry which considerably lower the detection limit. Nevertheless, the average expression measured on colonies by immunoblot allows detecting no-expression mutants among wild-type clones in which the expression of the phenotype is bimodal. Single cell level screening that could not be used as no-expression mutants would be included in the subpopulation of wild-type cells that transiently do not express the phenotype.

Hypothesis-driven phenotypic screening can constitute a relevant and affordable alternative. In principle, single cell level readout can be suited to detect and to sort clones that harbor mutations changing a phenotype otherwise homogeneously expressed in wild-type cells. If a specific fluorescent signal or cell shape can reveal the phenotype, it is possible to use a cell sorter to isolate relatively rare mutants that differ from the wild-type. However, phenotypic heterogeneity in the wild-type population may impair the detection of such mutants by single cell level approaches. This is particularly true for bimodal or bistable phenotypic expression. In this case, cells that share the same wild-type genome form two distinct subpopulations in a given environment, one expressing the phenotype (ON), the other not (OFF). This second population makes the detection of mutant cells genetically unable to express the phenotype difficult. A simple solution is to change the scale of analysis from the single cell to the clonal population level (e.g., isolated colonies) in which a no-expression mutant should be distinguishable from clonal populations presenting the wild-type average expression of the phenotype (**Figure 1**).

Phenotypic analysis at the colony level using an immunoblot assay has been used initially to screen for specific protein expression in defined strains (Henning et al., 1979; Mutharia and Hancock, 1985) or from recombinant protein libraries (Cornvik et al., 2005). Moreover, this has been used to identify bacterial pathogens within samples (Hull et al., 1993; Takai et al., 1994; Belyi et al., 1995; Hoszowski et al., 1996; Szakal et al., 2001, 2003; Bogaert et al., 2004; Chen and Ding, 2004; Huang et al., 2016). We recently derived a colony immunoblot assay to follow the evolution of virulence in populations of *Salmonella enterica* serovar Typhimurium (*S*. Typhimurium) (Diard et al., 2013). In this bacterium, the expression of the Type Three Secretion System-1 (TTSS-1) is bimodal and associated with a substantial growth defect (Hautefort et al., 2003; Sturm et al., 2011). We therefore predicted that fast-growing attenuated mutants should emerge during within-host growth. This hypothesis was indeed verified by using a colony immunoblot assay to monitor the average expression of TTSS-1 in clones obtained from fecal pellets of long-term infected mice (Diard et al., 2013).

Here, we present an optimized version of the colony immunoblot assay (ColoBlot) and a dedicated semi-automated open-source image analysis pipeline [Colony Immunoblot Image Analysis (CIIA)] available at https://sourceforge.net/projects/coloblot-image-analysis/files/. This protocol allows identification of phenotypic variants according to the intensity of the immunoblot signal. As proof of principle, we used this protocol to analyze evolved populations of *S*. Typhimurium from infected mice. We were able to select and to characterize clones expressing TTSS-1 at various levels. The ColoBlot could estimate the proportion of mutants expressing TTSS-1 at intermediate levels within mixed populations that also included wild-type strains and no-expression mutants. The ColoBlot, coupled with genomic analysis revealed a more subtle picture of the evolutionary dynamics of *S*. Typhimurium during infection than previously described (Diard et al., 2013).

## Materials and Methods

### Bacterial Strains

Strains used in this study are summarized in **Table 1**. All *S.* Typhimurium strains are derivatives of SL1344 (Hoiseth and Stocker, 1981). Bacteria were cultivated in Luria-Bertani (LB) medium containing the appropriate antibiotics [6 μg.ml^-1^ chloramphenicol (AppliChem); 50 μg.ml^-1^ kanamycin (AppliChem); 50 μg.ml^-1^ streptomycin (AppliChem)]. To construct gene deletion mutants, the targeted gene was replaced by an antibiotic resistance cassette using λ/red homologous recombination (Datsenko and Wanner, 2000). Desired mutations (or the P*prgH-gfp* reporter construct) were transferred into different genetic backgrounds by P22 HT105/1 *int-201*-mediated transduction (Sternberg and Maurer, 1991), and the antibiotic resistance cassettes were subsequently removed when needed by a temperature-inducible flippase encoded on pCP20 (Datsenko and Wanner, 2000). Either *cat* or *aphT* cassettes inserted into or between pseudogenes [*marT*::*cat*; *aphT*, between *malX* and *malY* (Grant et al., 2008)] conferred *in vivo* fitness neutral antibiotic resistances and were used for replica plating experiments.

**Table 1:** Bacterial strains.

Strain name	Strain number	Relevant genotype	Resistance	Reference
SL1344	SB300	Wild-type	Sm	Hoiseth and Stocker, 1981
SL1344 *sseD*::*aphT*	M556	*sseD*::*aphT*	Sm, Kan	Hapfelmeier et al., 2005
SL1344 Δ*hilD*	M3101	Δ*hilD*	Sm	Diard et al., 2013
SL1344 Δ*hilC*	Z1698	Δ*hilC*	Sm	This work
M556 SipC (+)		*sseD*::*aphT*	Sm, Kan	This work
M556 SipC (+/-)		*sseD*::*aphT hilC^∗^*	Sm, Kan	This work
M556 SipC (-)		*sseD*::*aphT hilD^∗^*	Sm, Kan	This work
SL1344 P*prgH*::*gfp*		P*prgH-gfp*	Sm, Cm	Hautefort et al., 2003
SL1344 Δ*hilD* P*prgH*::*gfp*	M3138	Δ*hilD*, P*prgH-gfp*	Sm, Cm	Diard et al., 2013
SL1344 Δ*hilC* P*prgH*::*gfp*	M3196	Δ*hilC*, P*prgH-gfp*	Sm, Cm	This work
M556 SipC (+) P*prgH*::*gfp*		*sseD*::*aphT*, P*prgH-gfp*	Sm, Cm	This work
M556 SipC (+/-) P*prgH*::*gfp*		*sseD*::*aphT, hilC^∗^*, P*prgH-gfp*	Sm, Cm	This work
M556 SipC (-) P*prgH*::*gfp*		*sseD*::*aphT, hilD^∗^*, P*prgH-gfp*	Sm, Cm	This work
SL1344 Cm^R^	M3020	*marT::cat*	Sm, Cm	Diard et al., 2014
SL1344 Δ*hilD* Kan^R^ Cm^R^	Z2078	*hilD*::*cat, aphT*	Sm, Cm, Kan	This work
SL1344 Δ*hilC* Kan^R^	Z2079	Δ*hilC, aphT*	Sm, Kan	This work
*Salmonella enteritidis*	P125109	Wild-type	None	Suar et al., 2009

^∗^*Mutated allele*.

### Optimized Colony Immunoblot

**FIGURE 2:**
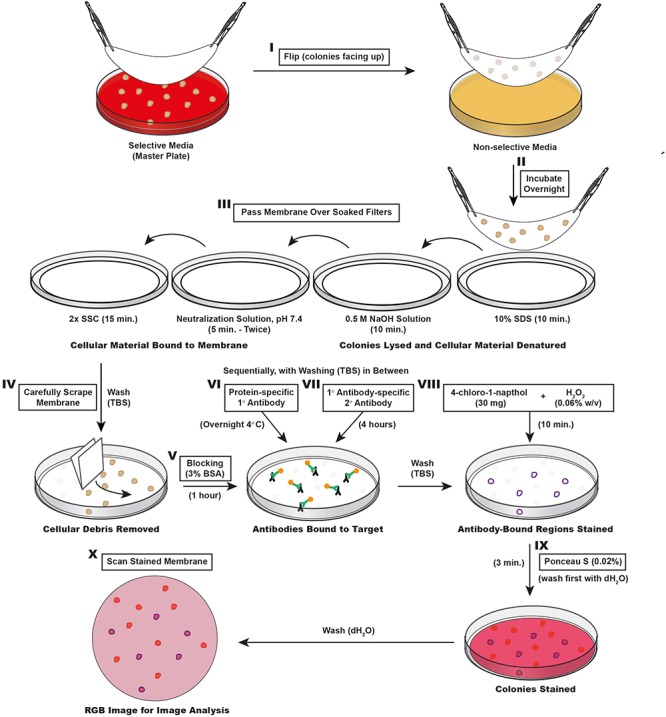
Schematic outline of the ColoBlot procedure. **(I)** Colonies grown on the master plate are transferred on a nitrocellulose membrane. **(II)** The membrane is then placed colony side up on a non-selective agar plate and incubated overnight. **(III)** The membrane is passaged over different buffer soaked Whatman papers to lyse colonies and bind cellular material to the membrane. **(IV)** The membrane is washed and excess cellular debris is removed by lightly scraping the membrane with Whatman paper. **(V)** Another round of washing is followed by blocking in TBS buffer containing 3% BSA. **(VI)** Protein-specific primary antibodies are added to the membrane in blocking solution. **(VII)** After three rounds of washing, HRP-conjugated secondary antibodies are added in blocking solution. Another three rounds of washing removes antibody in excess. **(VIII)** The staining was performed by exposing the membrane to 4-chloro-1-naphthol in presence of H_2_O_2_. This reaction is terminated after 10 min by washing with dH_2_O. **(IX)** A non-specific counter stain is performed by incubating the membrane in the presence of a Ponceau S solution. Destaining (to increase the signal-to-background ratio) is achieved with sequential washing with dH_2_O. **(X)** After drying, the membrane is ready to be scanned as an RGB TIFF image for CIIA.

We have refined the ColoBlot protocol described by Qiagen and designed to screen several hundreds of isolated clones for specific protein expression (Qiagen, Hilden, Germany^1^). Compared to our previous work with this protocol (Diard et al., 2013), we present here a semi-quantitative analysis pipeline to detect subtle differences in protein expression levels on colonies. This is a broadly applicable method that we employed to detect SipC protein abundance at the clonal level (**Figure 2**) and identify *Salmonella* spp. based on their O serotype (**Figure 8**).

Master plates are obtained by plating appropriate dilutions of bacterial suspensions on selective media (we used MacConkey agar in 85 mm diameter Petri dishes to select for *S.* Typhimurium or *S. enterica* serovar Enteritidis [strain P125109 (Suar et al., 2009)] upon incubation at 37°C overnight). For a reliable image analysis, we recommend aiming for a maximum population of about 250 colonies per plate (given that *S*. Typhimurium forms 2–3 mm diameter colonies in these conditions). Also, prefer bead plating to Drigalski spatula for homogenous distribution of the colonies all over the plate.

A circular nitrocellulose membrane (Whatman Protran nitrocellulose membranes, 0.45 μm pore size, 82 mm diameter; Sigma) is carefully placed on the resulting colonies using two tweezers (placed center of membrane in the center of the master plate first). After covering the plate with the entire surface of the membrane, it is peeled from the master plate and flipped colony side up onto non-selective media (**Figure 2I**) and incubated (overnight at 37°C on LB agar for *S.* Typhimurium) (**Figure 2II**). It is important that the membrane is lifted carefully but swiftly to avoid splattering of colony material which makes identifying single clones difficult. The membrane is then treated by passages over Whatman papers (Whatman 3MM chromatography and blotting paper; Sigma) soaked with 10% SDS for 10 min, denaturation solution (0.5 M NaOH, 1.5 M NaCl) for 10 min (note that for *Salmonella* O side-chain staining, the denaturation step is not used as the alkaline pH denatures the O-acetylation which constitutes the O5 epitope), neutralization solution (1.5 M NaCl, 0.5 M Tris–HCl, pH 7.4) for 5 min twice, and 2× SSC (3 M NaCl, 0.3 M sodium citrate, pH 7) for 15 min (**Figure 2III**). Note that it is important that Whatman papers are thoroughly and evenly soaked, and that excess liquid has been removed. Moreover, membranes must be placed onto Whatman papers slowly to avoid formation of air bubbles. We have observed, nevertheless, that colonies on the edges of the membrane are often heterogeneously stained. This can be due to an excess of buffer on the filters, which washes proteins away from the membrane on the edges. This should be taken into consideration when analyzing membranes.

For the next steps, the membrane is placed in an empty 85 mm Petri dish. The membrane is washed with Tris-buffered saline (TBS; 10 mM Tris–HCl, 150 mM NaCl, pH 7.5) for 15 min by shaking on a rocking platform. The remaining cellular debris are then removed by scraping the surface of the membrane with Whatman paper (**Figure 2IV**). Excess debris can interfere with antibody binding and Ponceau S staining. The scraped membrane is washed a second time with TBS for 15 min before blocking with 5 ml of 3% bovine serum albumin (BSA) in TBS for 1 h at room temperature with slow shaking (**Figure 2V**).

After blocking, 5 ml of the primary antibody diluted in 3% BSA TBS are added to the membrane (**Figure 2VI**). For SipC, we use a 1:5000 dilution of an antiserum provided by Virotech Diagnostics GmbH (reference number: VT110712). For *Salmonella* O5 antigen, we use a 1:1000 dilution of *Salmonella* O Antiserum Factor 5 (Difco; Cat. No. 226601). The membrane is then incubated on a rocking platform in a moist chamber overnight at 4°C. Washing in TBS-T (20 mM Tris–HCl, 500 mM NaCl, 0.05% Tween 20, and 0.2% Triton X-100, pH 7.5) for 10 min and in TBS twice more for 10 min removes non-specific primary antibody binding. A total of 5 ml of diluted horseradish peroxidase (HRP)-conjugated secondary antibody [we use a 1:1000 dilution of a Goat-anti-rabbit IgG HRP antibody (LabForce, reference number: sc-2004) in 3% BSA TBS] are then added to the membrane before incubation on a rocking platform for 4 h at room temperature (**Figure 2VII**). Antibodies in excess are eliminated by washing the membrane three times 10 min in TBS.

The staining is revealed by the chromogenic substrate 4-chloro-1-naphthol (Sigma). A 30 mg tablet of 4-chloro-1-naphthol is dissolved in 10 ml of methanol and mixed with H_2_O_2_ (0.06% w/v) in 50 ml TBS. A total of 5 ml of this solution is added per membrane (**Figure 2VIII**). After 10 min incubation at room temperature on a rocking platform, the reaction is stopped by washing the membrane with dH_2_O. To counterstain the colonies with the non-specific Ponceau S red stain, the membrane is incubated for 3 min at room temperature in a Ponceau S solution (0.02% w/v Ponceau S in 1% v/v acetic acid; **Figure 2IX**). The staining in excess is then removed with dH_2_O by washing the membrane four times for 5 min each on a rocking platform (or until colonies are clearly distinguishable from the background). The Ponceau S staining is the modification of the protocol used in Diard et al. (2013) that allows automated detection of regions of interest (ROI).

The membrane is then allowed to dry and scanned (Canoscan LiDE 700F, Canon) as a high-quality RGB image (TIFF files are required for batch processing) (**Figure 2X**). However, too long free drying of the membrane can provoke wrinkles, which interferes with maximum intensity measurements. It is therefore recommended to store the membranes between two plastic sheets, and away from sunlight to avoid bleaching. Note that the membranes can be stored prior to Ponceau S staining, which can be performed at any time. If ROI detection is suboptimal due weak to Ponceau S staining, membranes can be dried and re-stained with a higher concentration Ponceau S solution.

### Semi-automated Image Analysis for Phenotypic Classification

**FIGURE 3:**
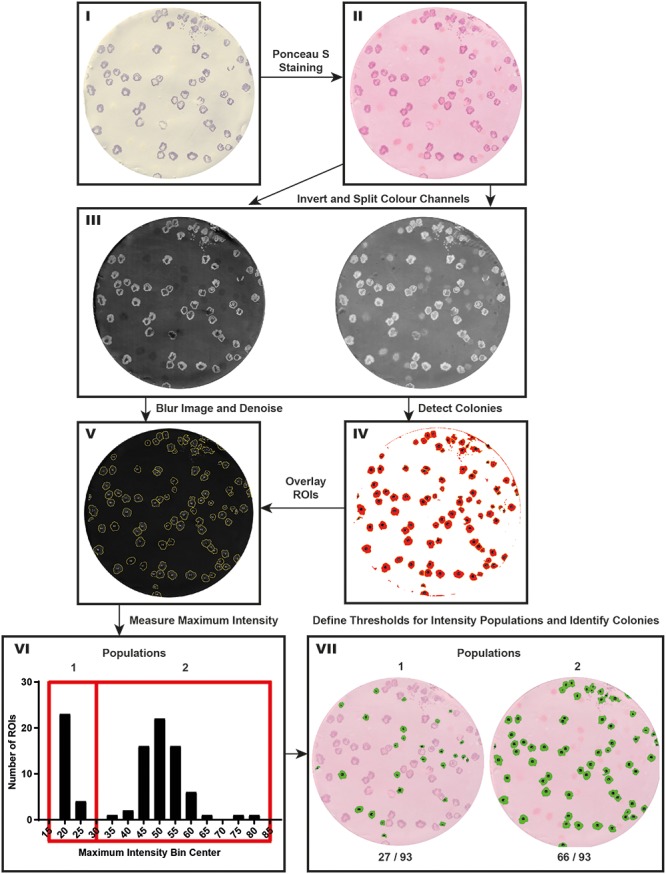
The CIIA pipeline. **(I)** The ColoBlot procedure was performed on a plated mixture of SL1344 (wild-type) and SL1344 Δ*hilD* using an anti-SipC primary antibody. **(II)** Colonies that did not express SipC, and are therefore not stained by 4-chloro-1-naphthol, are made visible by the non-specific Ponceau S staining. **(III)** The image (from **II**) is split into its color component images [left membrane: red color component image (SipC stain); right membrane: green color component image (Ponceau S stain)]. **(IV)** After thresholding (colony detection threshold = 61), binary masking, and binary operations, ROIs are identified by the Ponceau S stain based on parameters inputted by the user (Analyze Particles). **(V)** The image of the SipC staining is blurred and denoised, and the ROIs (detected in IV) are overlain. **(VI)** The maximum intensity is measured for each ROI and plotted as a histogram (shown histogram was produced with GraphPad Prism version 7 for windows using maximum intensity data saved in the XLS file by CIIA). A threshold (here 30) is manually selected based on local minima in the histogram. **(VII)** ROIs are classified and highlighted on the output image by CIIA, based on the threshold definition (identified in **VI**).

In order to create an unbiased method for quantification of protein content using ColoBlot, we developed a simple, user-friendly ImageJ (Fiji) macro (**Figure 3**). This macro (CIIA.txt^2^) allows for the unbiased detection and relative quantification of clone staining resulting from the ColoBlot procedure. The CIIA pipeline consists of three major steps: (1) identifying regions on the membranes where cellular material from colonies has been stained; (2) quantifying the staining intensity on each of these colonies; and (3) identifying clones according to phenotypic characteristics using thresholds defined by the user. A step-by-step description of how to use the macro as well as troubleshooting information is contained within a readme.txt file.

In order to identify regions on the membrane where colonies have grown, a red Ponceau S stain is used, which binds protein (**Figure 3II**). The chromogenic substrate used (4-chloro-1-naphthol) yields a dark purple hue of intensity proportional to the amount of the primary antibody target. Therefore, after splitting a RGB image [600 dots per inch (dpi) TIFF files are recommended] into its color components (red, green, and blue), colonies can be identified by the red stain, and the specific target by the purple stain. This macro requires an image to be open with Fiji^3^.

To facilitate object detection, the image is inverted to yield bright regions of interest on a dark background prior to color component splitting. The green negative color component image is used to identify colonies and append ROIs to the ROI manager (**Figures 3III,IV**). The contrast of the component image is enhanced (Enhance Contrast), the image is blurred (Gaussian Blur), and a dialog box prompts the user to adjust a threshold to facilitate image segmentation (if manual thresholding is selected). Once the threshold is defined (automatic thresholding methods can also be used as specified in the parameter dialog box), the image is segmented and converted to a binary image. Processing the binary image assists in ROI identification. High intensity segmented regions are sequentially dilated (Maximum), gaps are closed (Close), interiors are filled (Fill Holes), and eroded back to their original size (Minimum), using similar binary processing operations as for the identification of colonies on an agar plate (Choudhry, 2016). In order to isolate colonies found in clusters (if this option is selected in the parameter dialog box), the watershed algorithm is used. ROIs are then detected (Analyze Particles) based on particle size (**Figure 3IV**) (default 2000–15000 pixels in area) specified in the parameter dialog box, and appended to the ROI manager.

The second main component of CIIA uses the identified ROIs to measure specific staining intensity. The red negative color component image is denoised (Despeckle) and blurred (Gaussian Blur), and the ROIs are overlaid from the ROI manager (**Figure 3V**). The maximum intensity of each ROI on the red component image is measured. The maximum intensity parameter was chosen since performing the ColoBlot procedure for SipC expression leads to a ring-shaped staining. Comparing mean or median intensity of ROIs with and without a ring would lead to falsely undetected differences. The macro saves these measurements as an XLS file [additional information (mean, minimum intensity, area) is also saved] and creates a histogram window, indicating the distribution of maximum intensities (**Figure 3VI**).

The last main component of the macro identifies clones that satisfy population-defining criteria inputted by the user. There are two main approaches to define thresholds: (1) prior information regarding the number of expected populations can allow for threshold definitions (**Figures 3VI**, **7**); and (2) a comparison with a reference population (e.g., ancestors) can allow for the statistical identification of clones that deviate phenotypically from the reference population (**Figure 5**). Once the number of populations (the macro allows up to 4) and thresholds are identified, this information is entered into the dialog box. Then, CIIA creates and saves a JPEG image containing ROI overlays of the clones of interest (**Figure 3VII**).

We have also created a second macro (CIIA_Batch.txt) that allows for batch processing of images that have similar thresholds for ROI detection and maximum intensity-based population definition. The method for thresholds definition is identical to the single image macro. Therefore, we recommend setting these thresholds according to CIIA on at least some representative images prior to using CIIA_Batch (see example in **Figure 7**). For each image analyzed by CIIA_Batch, an XLS file of ROI measurements is saved (yields the same values as CIIA, provided an identical ROI detection threshold). As quality control, for each image analyzed by CIIA_Batch, three JPEG files are saved: the color-coded output populations detected according to maximum intensity thresholds; the specific color component images with overlaid ROIs; and a snapshot of the maximum intensity histogram of detected ROIs. A TXT file of the log is saved which contains the defined parameters, thresholds used, and counts of ROIs in each population.

### Colony PCR

**FIGURE 4:**
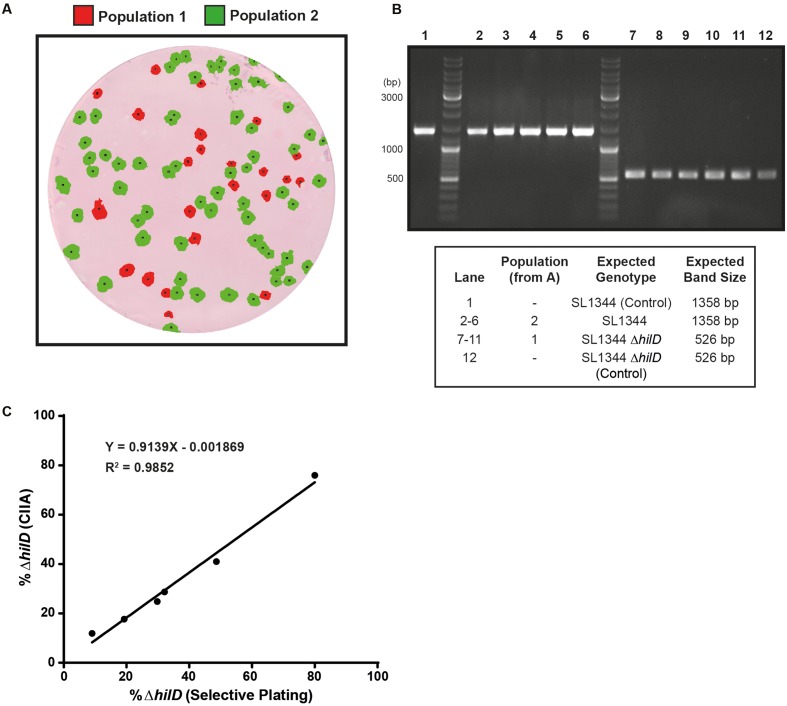
The CIIA can accurately identify Δ*hilD* mutants mixed with wild-type clones. Membranes containing mixtures of SL1344 (wild-type) and SL1344 Δ*hilD* were processed using CIIA_Batch (maximum intensity threshold = 40). **(A)** Representative image of population overlays (same image as shown in **Figure 3**). **(B)** Five clones of each population [identified in **(A)**] were tested for the expected genotype by colony PCR. **(C)** A regression analysis was performed to determine CIIA efficiency on six membranes containing mixtures of SL1344 (wild-type; Cm^R^) and SL1344 Δ*hilD* at different ratios, through comparison with selective replica plating. Slope = 0.9139 (extra sum-of-squares *F*-test compared to slope = 1 not significant; *p* = 0.1983); origin = –0.0001869; *R*^2^ = 0.9852.

To verify the genotype of selected clones based on the output from the ColoBlot analysis performed on a mixture of wild-type and Δ*hilD* mutants (**Figure 4**), we used the following primers: ver_hilD_up2 (TCTCGATAGCAGCAGATTAC) and ver_hilD_dw2 (CAGTATAAGCTGTCTTCCG). The conditions were as follows: sample denaturation 92°C, 5′; 35 cycles of denaturation (92°C, 30′′), annealing (55°C, 30′′), and elongation (72°C, 2′) steps; final elongation 7′ at 72°C.

### Assessment of CIIA Pipeline Efficiency by Replica Plating

Bacterial suspensions containing mixtures of strains [differentiable by their respective antibiotic resistance(s)] were plated on MacConkey agar supplemented with streptomycin (Sm) (master plate) to yield 50–250 colonies (all *S.* Typhimurium strains used are Sm^R^). For experiments with mixtures of *S.* Typhimurium and *S*. Enteritidis, bacterial suspensions were plated on MacConkey agar without antibiotics. The colonies were then transferred to a nitrocellulose membrane from the master plate for ColoBlot analysis. The same master plate was also used to replica transfer colonies onto MacConkey agar containing appropriate antibiotics to enumerate *S.* Typhimurium wild-type (Cm^R^), Δ*hilD* (Kan^R^, Cm^R^), and Δ*hilC* (Kan^R^) colonies. Percentage of each genotype determined by replica plating were plotted against percentage of each genotype determined by CIIA and a linear regression was performed to assess correlation of results produced by the two techniques.

### Whole Genome Sequencing

For whole genome sequencing, pure cultures of selected clones were grown in LB supplemented with appropriate antibiotics and genomic DNA was extracted using the Qiagen DNeasy extraction kit. The Illumina MiSeq system operated at the Functional Genomic Center of Zürich was used to generate 250 bp paired-ends reads. The genome coverage was at least 50 times. Bioinformatics analysis was performed with CLC Genomic workbench 6.5.1. Reads were assembled, and single nucleotide polymorphisms and small insertions/deletions were detected using the sequenced genome of *S*. Typhimurium SL1344 as reference (Hoiseth and Stocker, 1981; Kroger et al., 2012).

### Flow Cytometry

*Salmonella Typhimurium* strains expressing green fluorescent protein (GFP) under the control of the *prgH* promoter (Hautefort et al., 2003) were grown overnight at 37°C in LB with the appropriate antibiotics, diluted 1/20 and sub-cultivated for 4 h in LB without antibiotic. Cells were washed and diluted 1/10 in PBS before flow cytometry analysis. GFP emission per cell was determined using a LSRII flow cytometer (BD Biosciences) and analyzed with FlowJo software (Tree Star).

### Animal Experiments

Experimental *in vivo* evolution and within-host competitions were performed in 9–12 weeks old C57BL/6 or 129 SvEv mice pretreated with streptomycin as described earlier (Barthel et al., 2003). Mice were maintained under specified pathogen-free conditions in individually ventilated cages at the RCHCI facility of ETH Zürich. The experiments were approved by the responsible administration (Tierversuchskommission, Kantonales Veterinäramt Zürich, licenses 222/2013 and 193/2016).

### Biosafety

All experiments were performed by trained personal in biosafety level 2 laboratories (HCI building of the ETH Zurich, Hoenggerberg) in accordance with standard BSL2 working procedures.

### Statistics

Statistical tests were performed using GraphPad Prism version 7 for windows^4^.

## Results

### Detection of Clones That Do Not Express TTSS-1

The first step was to establish the ColoBlot protocol (**Figure 2**) and the image analysis pipeline (**Figure 3**), as detailed in the section “Materials and Methods.” We improved the SipC colony immunoblot previously used to detect mutants that failed to express TTSS-1 in evolving populations of *S*. Typhimurium (Diard et al., 2013). The SipC protein is a secreted translocator located at the tip of TTSS-1. The expression of SipC can therefore be used as a proxy to monitor the expression of the whole secretion system. Genome sequencing of SipC negative clones revealed mutations in the *hilD* gene that encodes the main transcriptional regulator of TTSS-1 expression (Schechter et al., 1999; Diard et al., 2013).

We analyzed artificial mixtures of wild-type *S*. Typhimurium and Δ*hilD* mutants, which, respectively, do or do not express SipC (**Figure 4**). Accordingly, the distribution of the SipC staining signal yielded two distinct populations of colonies corresponding to the two genotypes (**Figure 4**). After defining thresholds to differentiate populations based on maximum SipC-intensity distributions, Δ*hilD* and wild-type clones could be spotted by CIIA (**Figures 3**, **4A**). The genotype of five clones belonging to each population were verified by PCR amplification of the *hilD* locus. We were able to recover wild-type or Δ*hilD* genotypes with full accuracy (**Figure 4B**). To further evaluate the efficiency of the image analysis, we compared the percentage of Δ*hilD* clones calculated either using CIIA or replica plating from the same master plates inoculated with controlled mixtures of Δ*hilD* (Chloramphenicol sensitive; Cm^S^) and wild-type strains (Chloramphenicol resistant; Cm^R^) at varying ratios (**Figure 4C**). The two approaches yielded comparable results [linear regression slope = 0.9139 (not significantly different from a slope of 1 (*p* = 0.1983)), origin = 0.0019, *R*^2^ = 0.9852], i.e., Δ*hilD* mutants identified by the absence of growth on chloramphenicol plates after replica plating were also consistently detected by ColoBlot and CIIA among the wild-type colonies.

### Identification and Characterization of Mutants Emerging During Within-Host Evolution

**FIGURE 5:**
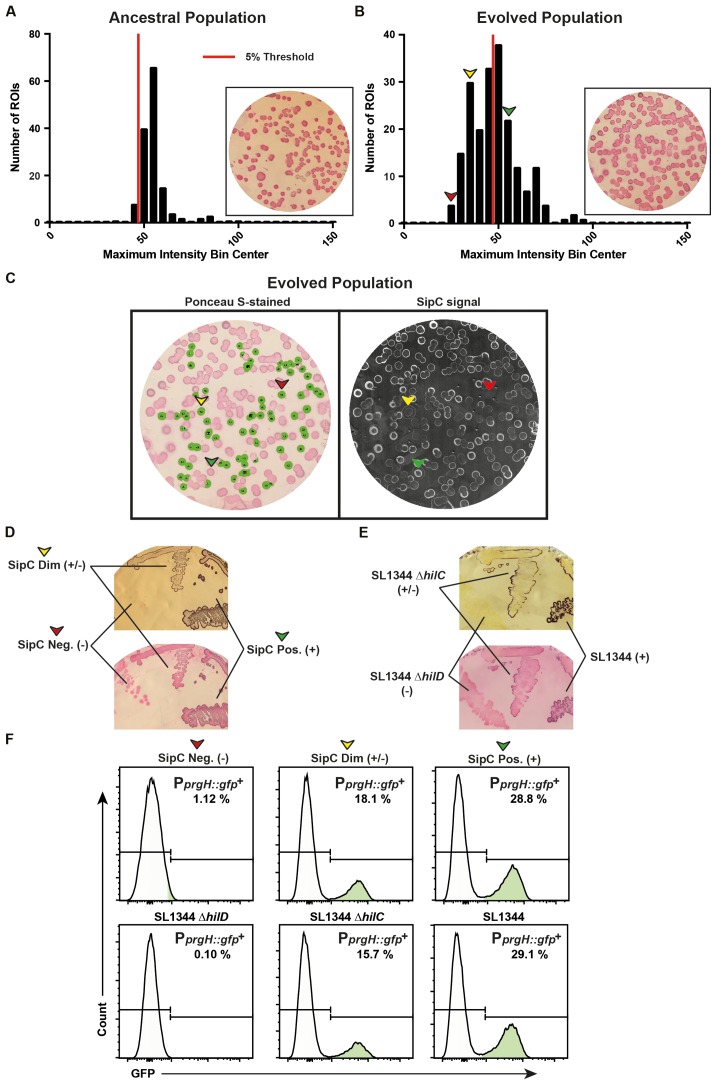
The ColoBlot analysis identifies TTSS-1 expression mutants emerging during within-host evolution. **(A–C)** M556 (Δ*sseD S.* Typhimurium SL1344; TTSS-1 expressing) were allowed to evolve for 10 days in C57BL/6 mice and fecal pellets were plated to yield 50–250 colonies per plate. The ancestral population (M556; plated after 4 days in C57BL/6) served as reference for SipC expression. An anti-SipC ColoBlot, followed by Ponceau S staining, was performed on the ancestral population (day 4 p.i.) and the evolved population (day 10 p.i.) [seen as insets in **(A,B)**, respectively]. **(A)** Maximum intensity histogram determined by CIIA of the ancestral population. The red line indicates the threshold corresponding to the upper value (47.0) of the first 5% of maximum intensity measurements (calculated by taking the maximum intensity value of ROI “*n*” that corresponds to the total ROI number multiplied by 0.05). **(B)** Maximum intensity histogram of the evolved population determined by CIIA. The 5% threshold of the ancestral population (determined in **A**) is overlaid. ROIs with high (+; green arrow), medium (+/–; yellow arrow), or low (–; red arrow) maximum intensity were selected based on ROIs above the threshold (expected ancestral genotype; green arrow), and ROIs at the upper (yellow arrow) and lower (red arrow) bounds of the maximum intensity distribution below the threshold. **(C)** Output image of the evolved population by CIIA, with ROIs below the threshold shown in green (left image). SipC color component image (right image). Clones expressing high amount (+; green arrow), low amount (+/–; yellow arrow), or no (–; red arrow) SipC are indicated (as identified in **B**). **(D)** Clones identified from **(B)**, and isolated from the master plate (membrane shown in **C**), were streaked onto a MacConkey plate and an anti-SipC ColoBlot was performed to confirm their respective SipC expression level. Upper image shows the anti-SipC staining. The lower image shows the same membrane counterstained with Ponceau S. **(E)** Reconstructed strains carrying mutations as identified in the evolved clones by sequencing and corresponding to high (+; wild-type), low (+/–; Δ*hilC*), or no (–; Δ*hilD*) SipC expression. The strains were streaked onto a MacConkey plate and an anti-SipC ColoBlot was performed (upper image) and subsequently Ponceau S stained (lower image). **(F)** A *gfp* reporter cassette for TTSS-1 expression (P*prgH*::*gfp*) was inserted by P22 phage transduction into clones identified in **(C)** and the reconstructed strains shown in **(E)**, and analyzed using flow cytometry. Bacterial cells were identified by side-scatter. Percentages of GFP-positive events were calculated for each plot by defining a threshold according to the basal fluorescence level detected in a Δ*hilD* mutant.

In order to assess the efficiency of the method in realistic infection conditions, we analyzed samples obtained from *in vivo* evolution experiments. C57BL/6 mice were infected with TTSS-1 expressing strains of *S*. Typhimurium (M556; a Δ*sseD* attenuated mutant). The Δ*sseD* genotype allows for long-term within-host evolution in sensitive mice [as described in (Diard et al., 2013)]. The ColoBlot analysis was performed on suspended fecal pellets diluted and plated on selective MacConkey agar to obtain about 250 *S*. Typhimurium clones (**Figure 5**).

The distribution of SipC expression levels among clones from the population at day 10 post-infection (evolved population; **Figure 5B**) was compared with the distribution obtained from the population at day 4 post-infection (“ancestral population” with only limited within-host evolution; **Figure 5A**). The distribution of the evolved population overlapped only partially with the distribution from the population at day 4 post-infection (**Figure 5B**), that is, some clones within the evolved population displayed SipC expression levels clearly reduced compared to the ancestral population. In order to quantify the amount of clones that deviated from the initial distribution of SipC protein abundance per colony, we fixed a threshold equal to the first 5% of empirical maximum intensity measurements (**Figures 5A,B**). Three evolved clones were isolated from the master plate that contained the evolved population (**Figure 5C**, colored arrows): one clone (+) yielded SipC expression expected to belong to the ancestral population; two additional clones were selected from the intensity histogram below the selected threshold, indicating that these significantly differ from the ancestral population. One was selected with higher maximum intensity (+/-) and one with lower maximum intensity (-) below the threshold. Due to intermembrane SipC-staining variability that depends on the number of colonies present (**Figure 7A**), we selected values clearly below the defined threshold, and not at the upper bound. The staining procedure was repeated after streaking these clones on new MacConkey plates. Differences in the intensity of the coloration were clearly visible between the three isolated clones (**Figure 5D**). The homogeneity of the signal along the streaks and within colonies suggested stable phenotypes resulting from distinct genotypes. The genomes of the isolated clones were therefore sequenced and compared to the ancestral genome. Dim (+/-) and negative (-) SipC expression were associated with single nucleotide polymorphisms in *hilC* and *hilD*, respectively. The *hilC* gene encodes a transcriptional regulator homologous to HilD and known to positively regulate TTSS-1 expression (Schechter et al., 1999). Reconstructed *hilC* and *hilD* knockout mutants displayed comparable SipC expression levels than their counterparts arising during within-host evolution (**Figure 5E**). These observations were confirmed by flow cytometry using a *gfp* reporter gene under the control of a HilD-regulated promoter of *prgH* inserted in the genetic background of isolated clones and reconstructed mutants (**Figure 5F**). These results shown that the ColoBlot analysis was sensitive enough to reveal evolved clones harboring mutations that lead to a slight reduction of the population size expressing TTSS-1, roughly twofold as determined by flow cytometry (**Figure 5F**).

### Optimization of the Identification and Quantification of Distinct TTSS-1 Expressing Clones

**FIGURE 6:**
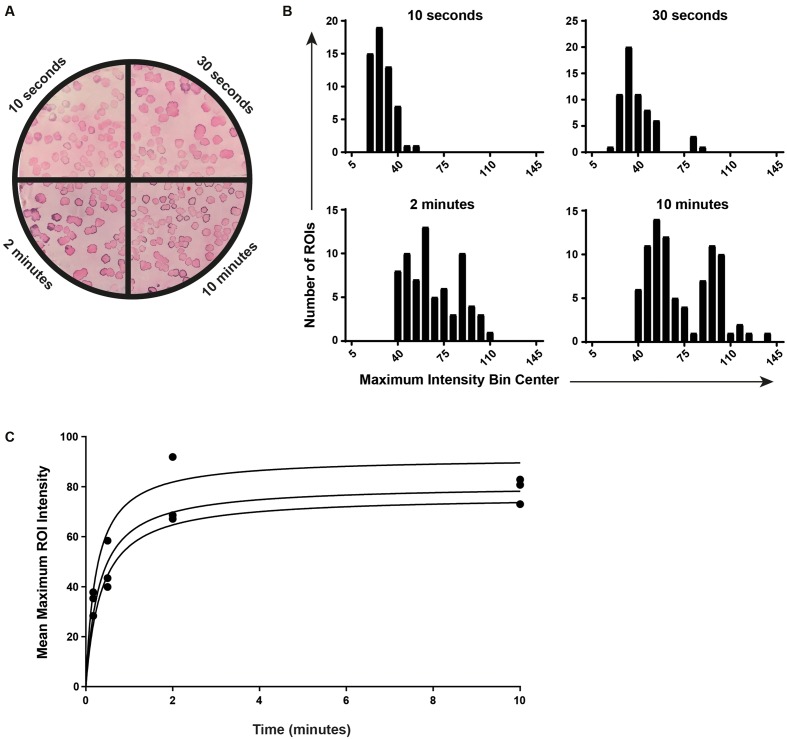
Optimization of the incubation time with the chromogenic substrate. **(A)** Equal mixtures of SL1344 Δ*hilC* and SL1344 wild-type were plated on MacConkey agar to obtain approximately 50–250 colonies. An anti-SipC ColoBlot was performed. Prior to exposure of the membrane to the 4-chloro-1-naphthol reagent mixture, the membrane was cut into four equal parts (representative images for each quadrant are shown divided by a black line). Each quadrant was exposed to 4-chloro-1-naphthol in the presence of H_2_O_2_ for 10 s, 30 s, 2 min, or 10 min (as indicated). **(B)** CIIA was performed on each quadrant independently and the resulting maximum intensity histograms are shown (representative histograms of the membrane shown in **A**). **(C)** The mean maximum intensity for each ROI (i.e., the mean maximum intensity for each histogram in **B**) in each quadrant is plotted as a function of time. Three membranes are plotted and hyperbolic curves are fit (*R*^2^ = 0.9119, 0.9703, and 0.931).

We next addressed the ability of CIIA to reproducibly detect small differences in SipC protein quantities per colony. We observed that depending on the overall intensity of the specific signal, identification of Δ*hilC* mutants in the presence of wild-type clones was not always accurate. Therefore, we tested the ability of CIIA to distinguish Δ*hilC* from wild-type on membranes incubated for increasingly long times in presence of the 4-chloro-1-naphthol chromogenic substrate (**Figure 6**). For incubation times longer than 2 min, the two distinct populations were more clearly discriminated (**Figures 6A,B**). We found that the mean of the maximum intensities of all ROI per membrane reached a plateau after about 2 min of incubation, suggesting that the reaction was limited by the amount of available substrate (**Figure 6C**). This may introduce a bias in the signal intensity of each ROI depending on the total amount of HRP molecules per membrane using the substrate. We henceforth use 10 min of incubation in presence of the substrate to resolve populations in complex mixtures of clones.

**FIGURE 7:**
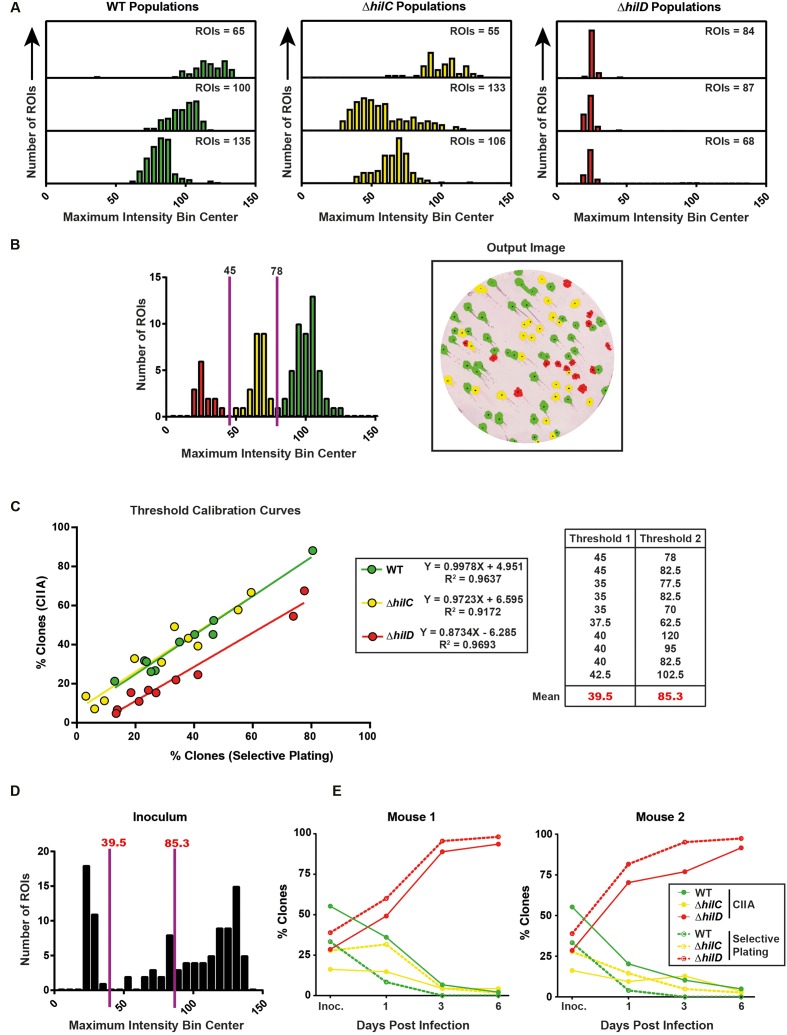
The ColoBlot analysis can be used to follow changes during within-host growth. **(A)** MacConkey master plates with 50–150 colonies containing only SL1344 wild-type (left histograms), SL1344 Δ*hilC* (middle histograms), or SL1344 Δ*hilD* (right histogram) were analyzed with the CIIA. Stacked histograms show maximum intensity measurements of three independent membranes for each population. The total number of ROIs in each histogram is indicated. **(B,C)** SL1344 wild-type (Cm^R^), SL1344 Δ*hilD* (Cm^R^; Kan^R^), and SL1344 Δ*hilC* (Kan^R^) were mixed at different ratios and plated on MacConkey agar to yield 50–250 colonies. The CIIA was used to quantify expression of SipC. **(B)** A representative histogram of one membrane is shown. The thresholds are manually determined to fit the local minima in the histogram (shown as solid purple lines; numbers indicate the threshold value) and the resulting populations on the membrane are shown (right panel; colored ROIs correspond to colored bars in the maximum intensity histogram). **(C)** Replica plating was performed in parallel to CIIA and a correlation analysis was performed. Linear regression best-fit equations and *R*^2^ values are shown in the panel inset. Wild-type: slope = 0.9978 [extra sum-of-squares *F*-test compared to slope = 1 not significant (*p* = 0.9756)], origin = 4.951, *R*^2^ = 0.9637; Δ*hilC*: slope = 0.9723 [extra sum-of-squares *F*-test compared to slope = 1 not significant (*p* = 0.7956)], origin = 6.595, *R*^2^ = 0.9172; Δ*hilD*: slope = 0.8734 [extra sum-of-squares *F*-test compared to slope = 1 not significant (*p* = 0.0500)], origin = –6.285, *R*^2^ = 0.9693. Each individual membrane was adjusted to correct the threshold for inter-membrane maximum intensity variation (thresholds shown in a table; the mean is calculated for each threshold and is used for subsequent analysis). **(D,E)** Streptomycin-pretreated 129 Sv/Ev mice were orally infected with mixtures of SL1344 wild-type (Cm^R^), SL1344 Δ*hilD* (Cm^R^; Kan^R^), and SL1344 Δ*hilC* (Kan^R^) at a 1:1:1 ratio. Population sizes were followed by plating resuspending fecal pellets on MacConkey plates supplemented with streptomycin and by performing replica plating in parallel to ColoBlot and CIIA (thresholds used are the mean values determined through threshold calibration in **C**). **(D)** The histogram from the inoculum (1:1:1 ratio) is shown with indicated thresholds (shown as purple lines; numbers indicate threshold values). **(E)** Percentages of clones of each genotype (indicated by the legend; green = wild-type; yellow = Δ*hilC*; red = Δ*hilD*) detected after performing CIIA (solid lines) or replica plating (dashed lines) on plated resuspended fecal pellets (50–250 colonies). Fecal pellet population composition from two representative mice is shown.

We then tested the sensitivity and efficiency of the ColoBlot analysis on mixtures of wild-type (Cm^R^), Δ*hilC* (kanamycin resistant; Kan^R^), and Δ*hilD* (Cm^R^ and Kan^R^) mutants at various known ratios. The results from replica plating served as reference for CIIA accuracy (**Figure 7**). We generated unique thresholds for SipC expression that are characteristics of each population. We first assumed that all three strains should yield reproducible distributions of SipC expression levels. To test this, we subjected each strain plated on MacConkey agar (50–250 clones) to the ColoBlot analysis in triplicate (**Figure 7A**). Notably, depending on the number of ROIs present on each membrane, the intensity of the SipC staining was variable (**Figure 7A**). Therefore, it was necessary to set thresholds to the local minima of histograms from of a mixture of each population (**Figure 7B**). We performed such a threshold calibration on 10 mixtures of the three strains (each at different ratios) and compared the ability of CIIA to distinguish populations with the results of the replica plating method (**Figure 7C**). Clones of each strain were successfully identified using both methods as determined through linear regression (wild-type: slope = 0.9978 [not significantly different from slope of 1 (*p* = 0.9756)], origin = 4.951, *R*^2^ = 0.9637; Δ*hilC*: slope = 0.9723 [not significantly different from slope of 1 (*p* = 0.7956)], origin = 6.595, *R*^2^ = 0.9172; Δ*hilD*: slope = 0.8734 [not significantly different from slope of 1 (*p* = 0.0500)], origin = -6.285, *R*^2^ = 0.9693). Thus, we could calculate mean thresholds: threshold 1 (39.5) defining the boundary between Δ*hilC* and Δ*hilD* and threshold 2 (85.3) defining Δ*hilC* and wild-type.

### The Relative Proportion of Clones Displaying Distinct TTSS-1 Expression Profiles Can Be Followed in *in Vivo*-Evolved Populations

Finally, we studied competitions between wild-type, Δ*hilC*, and Δ*hilD* mutants in mice. The mean thresholds calculated by calibration fitted with the local minima in the distribution of maximum intensity values of colonies from the inoculum in which the three strains were mixed at close relative proportions (**Figure 7D**). We followed the evolution of these proportions during infection and compared results from the ColoBlot analysis with selective plating (**Figure 7E**). The dynamics revealed by the ColoBlot procedure could be indeed confirmed by selective plating. The Δ*hilD* mutant outcompeted the wild-type strain and the Δ*hilC* mutant.

Therefore, upon calibration of optimal thresholds, the ColoBlot analysis is suitable to estimate the relative proportion of populations of *Salmonella* evolving within-host according to the expression of TTSS-1.

## Discussion

We combined immunostaining and a Ponceau S counterstain that, coupled with the CIIA ImageJ macro, allows for phenotypic analysis of bacterial populations at the level of the colony. We show that this is particularly useful for the detection of mutants presenting phenotypes that are otherwise within the range of phenotypic variation observed at the single cell level in wild-type populations (**Figure 1**). To our knowledge, this is the first pipeline for the evaluation of protein expression level on colonies that is able to identify distinct clonal populations, to estimate their relative proportion, and to flag clones of interest for further isolation and characterization.

In order to implement the ColoBlot protocol, two criteria must, however, be met:

(1) This phenotypic readout relies on availability of antibodies directed against a specific target. Here we used antibodies recognizing SipC to reveal the expression of TTSS-1 and co-expressed genes in *S*. Typhimurium.
(2) The phenotype must be expressed *in vitro* (growth on agar-based media is essential for the ColoBlot protocol).

Moreover, the accuracy of the image analysis depends on the quality of the membrane and of the staining. Too many clones per membrane reduces the precision of the ROI detection. We therefore recommend an upper limit of 250 clones for *S*. Typhimurium-like colonies on 85 mm diameter plates. Folding and scratching of the membrane should also be avoided.

Insufficient removal of cellular debris that can shield the target of the primary antibody may render the specific signal deceptively heterogeneous. The Ponceau S counterstain constitutes a good quality-control for this as it clearly reveals cellular debris that stick to the membrane (data not shown).

As shown in **Figure 6C**, the chromogenic reaction is limited by the amount of available substrate for HRP. This means that the signal intensity per positive clone depends on the number of positive clones per membrane. High amounts of colonies expressing the target implies more HRP-conjugated secondary antibodies per membrane and a substrate concentration that may become limiting. This is likely the reason why maximum intensity measurements from three membranes containing clones from the same ancestral population resulted in variable distributions (**Figure 7A**). Indeed, membranes that contained fewer colonies consistently yielded higher maximum intensity measurements than membranes containing more clones from the same population. A calibration using defined mixed populations is therefore useful in order to calculate maximum intensity thresholds used for characterized mixed subpopulations of clones on several membranes (**Figure 7C**).

As a proof of concept, we used the ColoBlot to demonstrate that *S*. Typhimurium evolved during infection in mice toward establishment of mutants displaying various in TTSS-1 expression level. The conservative nature of the ColoBlot analysis allows recovering clones of interest from the master plate for further characterization. The sequencing of isolated clones’ genome identified point mutations in *hilC* that were responsible for a twofold reduction in the number of individual cells expressing TTSS-1 (confirmed by flow cytometry data; **Figure 5**). Our previous study using a less advanced version of the colony immunoblot only revealed the emergence of mutants that were not expressing TTSS-1 at all (i.e., *hilD* mutants) during within-host growth. Results presented here reveal a more complex scenario than previously proposed in order to describe the long-term evolutionary dynamics of *S*. Typhimurium within-host (Diard et al., 2013). Clones expressing TTSS-1 at wild-type level compete not only against *hilD*, but also against *hilC* mutants. We assume this to be linked to the fitness cost associated with TTSS-1 expression (Sturm et al., 2011; Diard et al., 2013).

**FIGURE 8:**
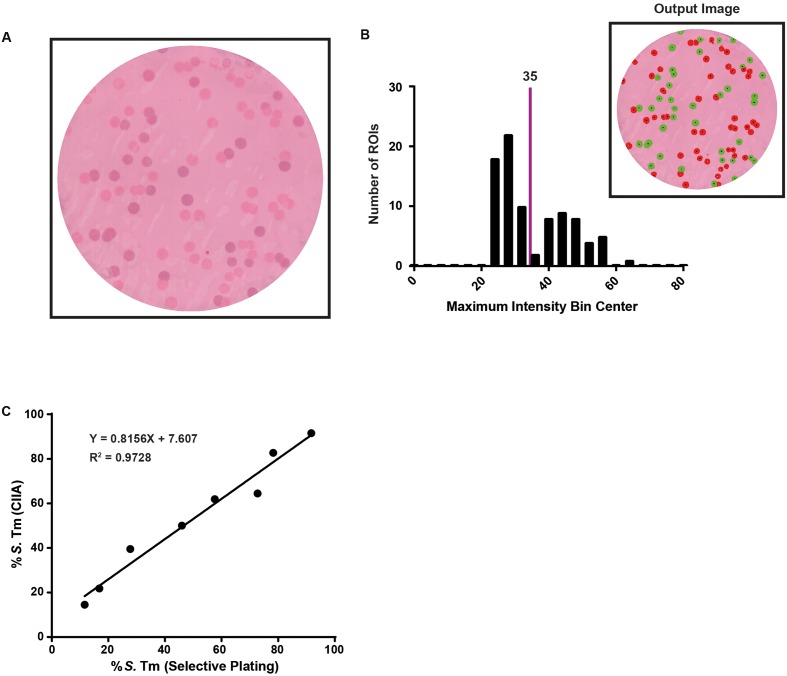
*Salmonella Typhimurium* and *S*. Enteritidis can be differentiated based on their O serotype using the ColoBlot analysis. **(A)** The ColoBlot procedure was performed on mixtures of *S.* Typhimurium (SL1344) and *S*. Enteritidis using a commercial anti-O5 serum as primary antibody. A representative image is shown stained with Ponceau S. **(B)** CIIA was performed on images from the ColoBlot procedure (see **A**) and a representative histogram is shown. A maximum intensity threshold (35) was determined based on the local minimum of the histogram. The colonies identified and classified by CIIA are shown in the inset (red indicates colonies below the threshold and green indicates colonies at or above the threshold). **(C)** Regression analysis comparing the CIIA output to selective replica plating from eight membranes containing mixtures of *S.* Typhimurium (SL1344; Cm^R^) and *S. Enterica* at different ratios. Slope = 0.8156 (extra sum-of-squares *F*-test compared to slope = 1; *p* = 0.0162); origin = 7.607; *R*^2^ = 0.9728.

The ColoBlot protocol is not limited to the study of within-host evolution. Changes in population composition can be monitored in any ecological niche providing that the bacteria of interest could be sufficiently enriched to obtain a reasonable amount of clones on selective agar medium. Furthermore, potential cellular targets of the primary antibody are not limited to proteins. As a proof of broad utility, we have used the ColoBlot pipeline to analyze the composition of *S.* Typhimurium and *S*. Enteritidis based on the *Salmonella* O antigen. The ColoBlot pipeline could accurately identify *S*. Typhimurium and *S*. Enteritidis based on the presence or absence of the O5 antigen, respectively (**Figure 8**). Distinct pneumococcal serotypes have also been identified using a colony immunoblot assay (Bogaert et al., 2004). Serotyping mixed populations has important implications in vaccine design and implementation. The ColoBlot pipeline can be used as an affordable alternative to flow cytometry analysis to monitor the evolution of pathogens upon vaccination using a polysaccharide-specific antibody. In conjunction with whole-genome sequencing, this could allow for the identification of novel classes of polysaccharide-modifying enzymes, and improve current mucosal vaccination strategies.

## Conclusion

The ColoBlot pipeline is a tool of potential broad applications, complementing single cell level approaches and whole-genome sequencing for the study of microbial population diversity.

## Author Contributions

EB and MD designed and performed the experiments. TD performed long-term mice infections. EB designed the image analysis ImageJ macro. EB and MD wrote the manuscript. All authors reviewed and commented on the manuscript.

## Conflict of Interest Statement

The authors declare that the research was conducted in the absence of any commercial or financial relationships that could be construed as a potential conflict of interest.

## Footnotes

1 https://www.qiagen.com/ch/resources/molecular-biology-methods/protein/

2 https://sourceforge.net/projects/coloblot-image-analysis/files/

3 https://fiji.sc/

4 http://www.graphpad.com
